# Supplementary material for: Spontaneous assembly of redox-active iron-sulfur clusters at low concentrations of cysteine
Source: Nat Commun. 2021 Oct 11;12:5925. doi: 10.1038/s41467-021-26158-2 (PMC8505563; doi:10.1038/s41467-021-26158-2)
Supplement: Supplementary file 1 — Supplementary Information [file 41467_2021_26158_MOESM1_ESM.pdf]

Supplementary Information

Spontaneous assembly of redox-active iron-sulfur clusters at low concentrations of cysteine

Sean F. Jordan[1], Ioannis Ioannou[1*], Hanadi Rammu[1]* Aaron Halpern[1], Lara K. Bogart[2], Minkoo Ahn[3], Rafaela Vasiliadou[1], John Christodoulou[3], Amandine Maréchal[3,4] and Nick Lane[1]

[1] *Centre for Life's Origin and Evolution, Department of Genetics, Evolution and Environment, Darwin Building, Gower Street, University College London, London WC1E 6BT, UK*

[2] *UCL Healthcare Biomagnetics Laboratory, University College London, 21 Albemarle Street, London, W1S 4BS, UK*

[3] *Institute of Structural and Molecular Biology, University College London, London, WC1E 6BT, UK*

[4] *Institute of Structural and Molecular Biology, Birkbeck College, London, WC1E 7HX, UK*

** These authors contributed equally to the work.*

Corresponding author: Nick Lane nick.lane@ucl.ac.uk

SI Results

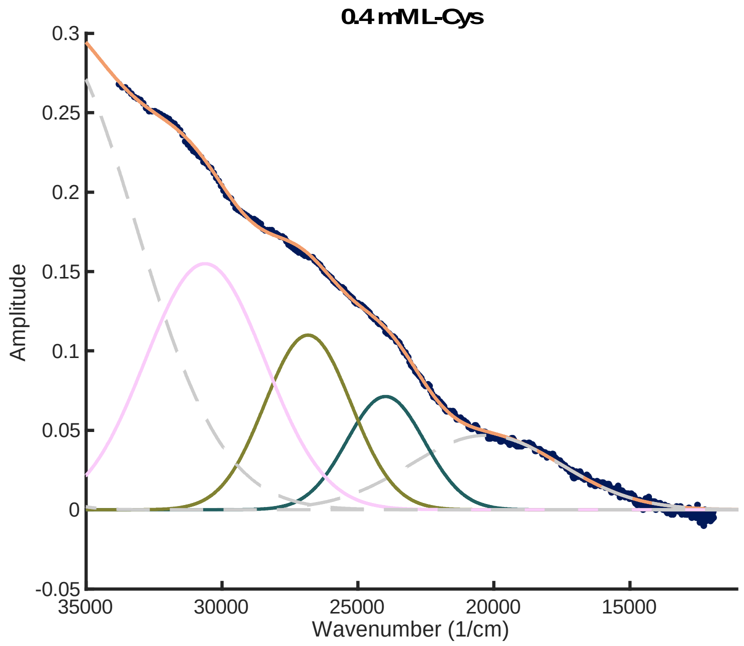

Supplementary Fig. 1. Plot of UV-Vis data for FeS clusters prepared with 0.4 mM L-cysteine in a 5:1:1 ratio with Fe and S. Fitting is based on the method of Galambas *et al.* (2019)[30] see also tutorials on https://zenodo.org/record/3533467#.YIsCAxNKjAM. Each peak corresponds to a specific excitation (From left to right: peak 1 grey dashed line – peptide charge-transfer; peak 2 grey dashed line – thiolate RS^- ⟶ Fe; peak 3 pink line – thiolate RS^- ⟶ Fe; peak 4 green line – S^{2-} ⟶ Fe; peak 5 blue line – S^{2-} ⟶ Fe; peak 6 grey dashed line – ligand field). Source data are provided as a Source Data file.

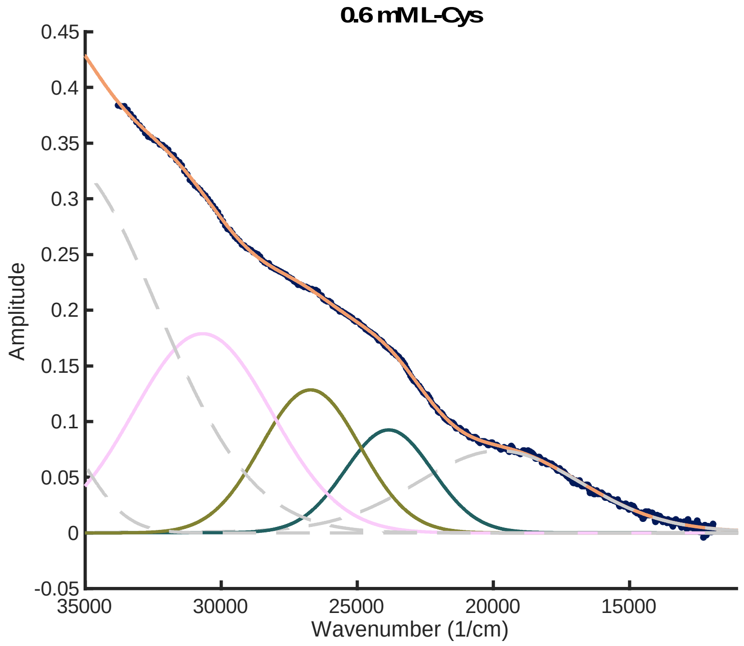

Supplementary Fig. 2. Plot of UV-Vis data for FeS clusters prepared with 0.6 mM L-cysteine in a 5:1:1 ratio with Fe and S. Fitting is based on the method of Galambas *et al.* (2019)[30]. Each peak corresponds to a specific excitation (From left to right: peak 1 grey dashed line – peptide charge-transfer; peak 2 grey dashed line – thiolate RS^- ⟶ Fe; peak 3 pink line – thiolate RS^- ⟶ Fe; peak 4 green line – S^{2-} ⟶ Fe; peak 5 blue line – S^{2-} ⟶ Fe; peak 6 grey dashed line – ligand field). Source data are provided as a Source Data file.

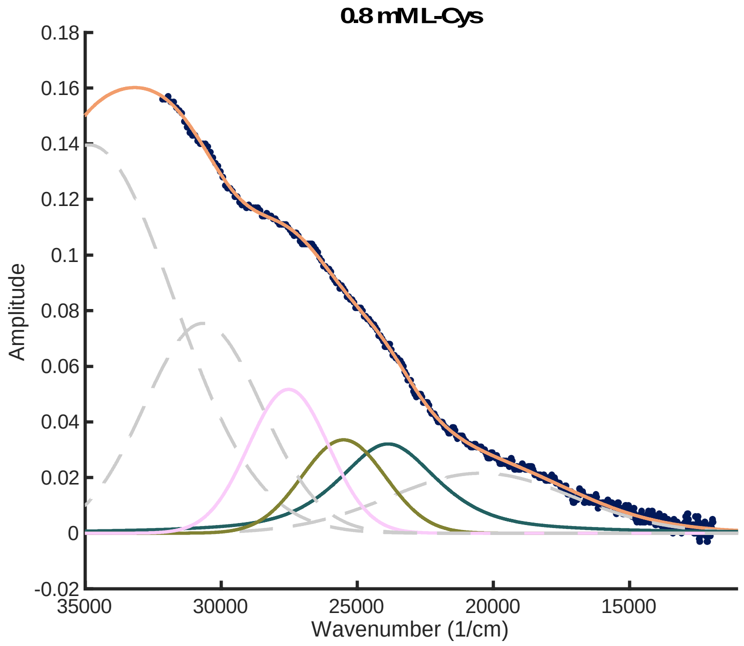

Supplementary Fig. 3. Plot of UV-Vis data for FeS clusters prepared with 0.8 mM L-cysteine in a 5:1:1 ratio with Fe and S. Fitting is based on the method of Galambas *et al.* (2019)[30]. Each peak corresponds to a specific excitation (From left to right: peak 1 grey dashed line – peptide charge-transfer; peak 2 grey dashed line – thiolate RS^- ⟶ Fe; peak 3 pink line – thiolate RS^- ⟶ Fe; peak 4 green line – S^{2-} ⟶ Fe; peak 5 blue line – S^{2-} ⟶ Fe; peak 6 grey dashed line – ligand field). Source data are provided as a Source Data file.

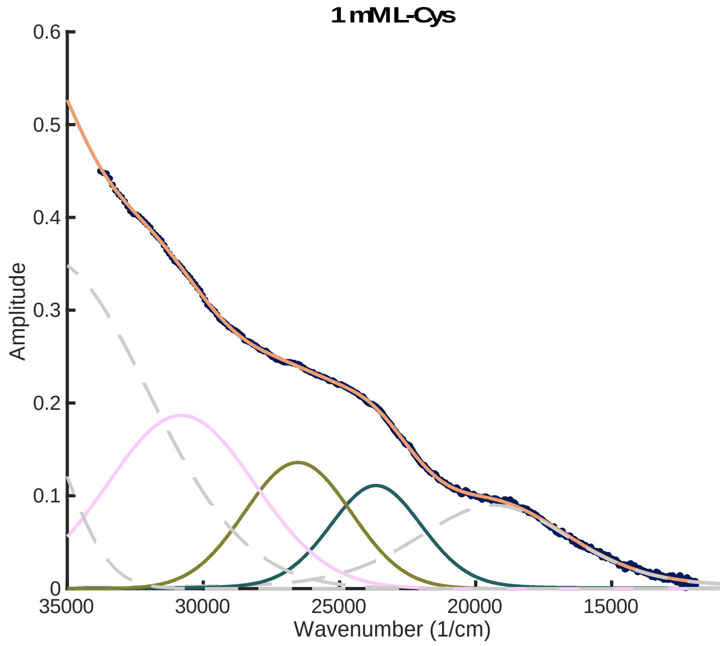

Supplementary Fig. 4. Plot of UV-Vis data for FeS clusters prepared with 1 mM L-cysteine in a 5:1:1 ratio with Fe and S. Fitting is based on the method of Galambas *et al.* (2019)[30]. Each peak corresponds to a specific excitation (From left to right: peak 1 grey dashed line – peptide charge-transfer; peak 2 grey dashed line – thiolate RS^- ⟶ Fe; peak 3 pink line – thiolate RS^- ⟶ Fe; peak 4 green line – S^{2-} ⟶ Fe; peak 5 blue line – S^{2-} ⟶ Fe; peak 6 grey dashed line – ligand field). Source data are provided as a Source Data file.

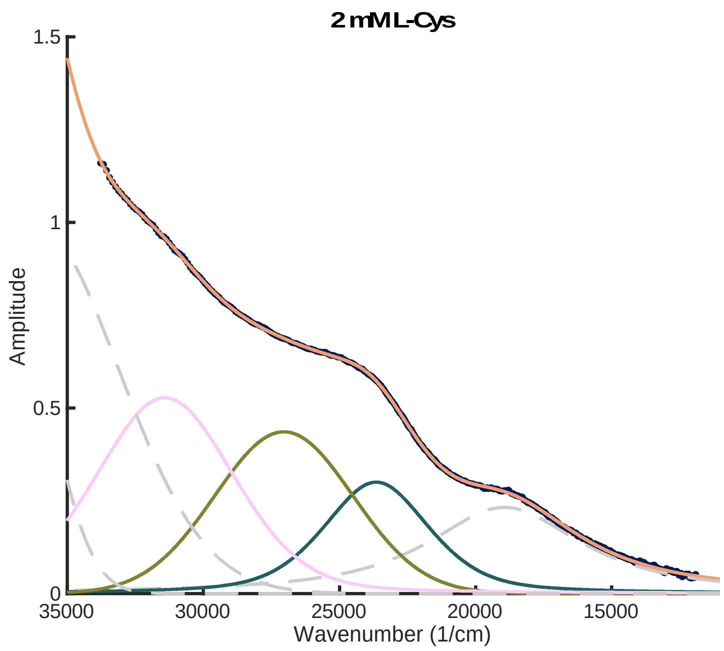

Supplementary Fig. 5. Plot of UV-Vis data for FeS clusters prepared with 2 mM L-cysteine in a 5:1:1 ratio with Fe and S. Fitting is based on the method of Galambas *et al.* (2019)[30]. Each peak corresponds to a specific excitation (From left to right: peak 1 grey dashed line – peptide charge-transfer; peak 2 grey dashed line – thiolate RS^- ⟶ Fe; peak 3 pink line – thiolate RS^- ⟶ Fe; peak 4 green line – S^{2-} ⟶ Fe; peak 5 blue line – S^{2-} ⟶ Fe; peak 6 grey dashed line – ligand field). Source data are provided as a Source Data file.

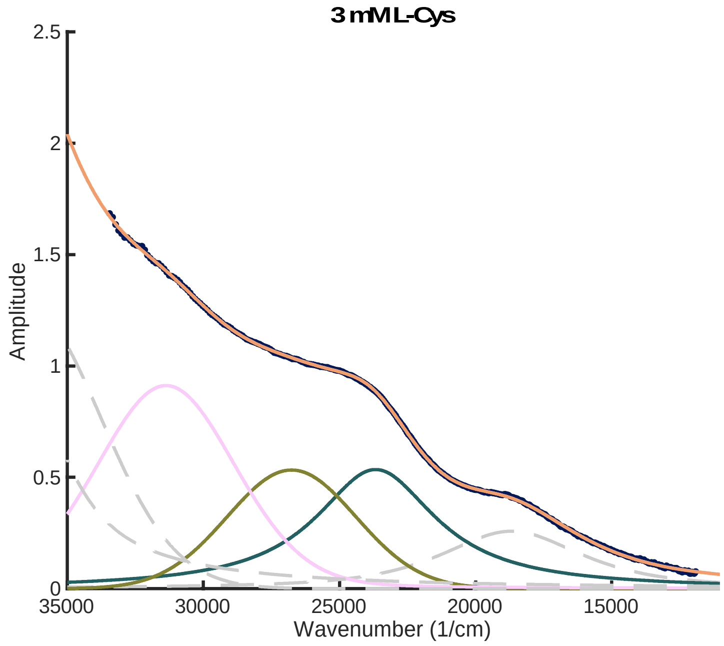

Supplementary Fig. 6. Plot of UV-Vis data for FeS clusters prepared with 3 mM L-cysteine in a 5:1:1 ratio with Fe and S. Fitting is based on the method of Galambas *et al.* (2019)[30]. Each peak corresponds to a specific excitation (From left to right: peak 1 grey dashed line – peptide charge-transfer; peak 2 grey dashed line – thiolate RS^- ⟶ Fe; peak 3 pink line – thiolate RS^- ⟶ Fe; peak 4 green line – S^{2-} ⟶ Fe; peak 5 blue line – S^{2-} ⟶ Fe; peak 6 grey dashed line – ligand field). Source data are provided as a Source Data file.

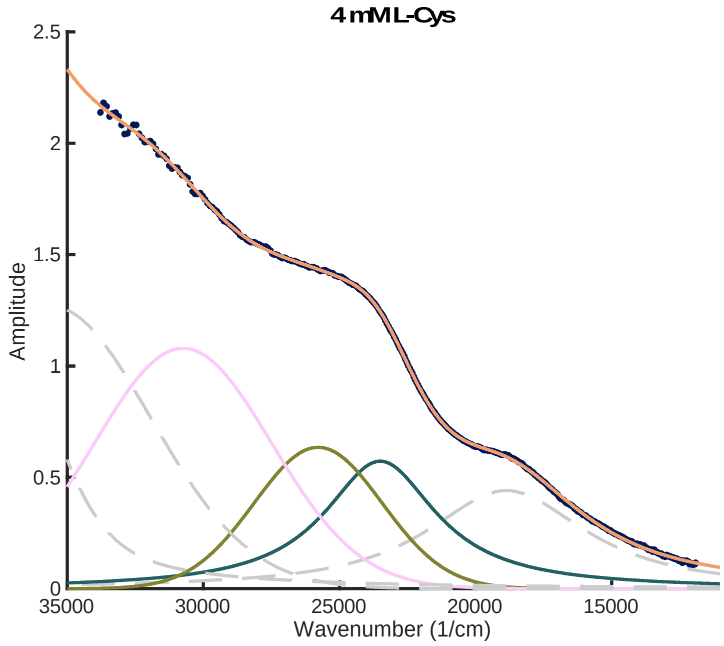

Supplementary Fig. 7. Plot of UV-Vis data for FeS clusters prepared with 4 mM L-cysteine in a 5:1:1 ratio with Fe and S. Fitting is based on the method of Galambas *et al.* (2019)[30]. Each peak corresponds to a specific excitation (From left to right: peak 1 grey dashed line – peptide charge-transfer; peak 2 grey dashed line – thiolate RS^- ⟶ Fe; peak 3 pink line – thiolate RS^- ⟶ Fe; peak 4 green line – S^{2-} ⟶ Fe; peak 5 blue line – S^{2-} ⟶ Fe; peak 6 grey dashed line – ligand field). Source data are provided as a Source Data file.

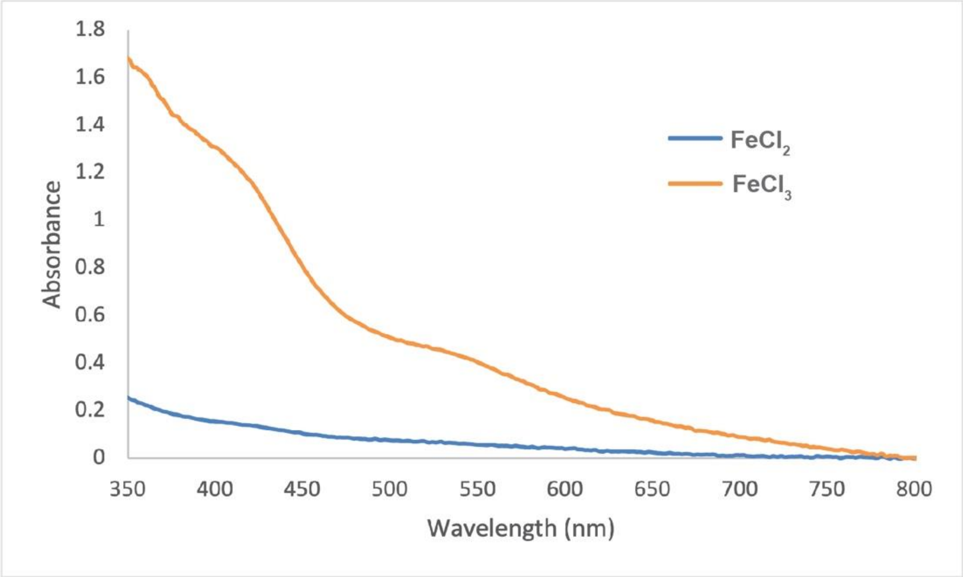

Supplementary Fig. 8. Representative UV-Vis spectrum for solution containing 5 mM L-cysteine, 1 mM $FeCl_2$ and 1 mM Na_2S at pH ~9 (blue line). Spectrum of solution prepared with 5 mM L-cysteine, 1 mM $FeCl_3$ and 1 mM Na_2S at pH 9 also included for reference (orange line). Source data are provided as a Source Data file.

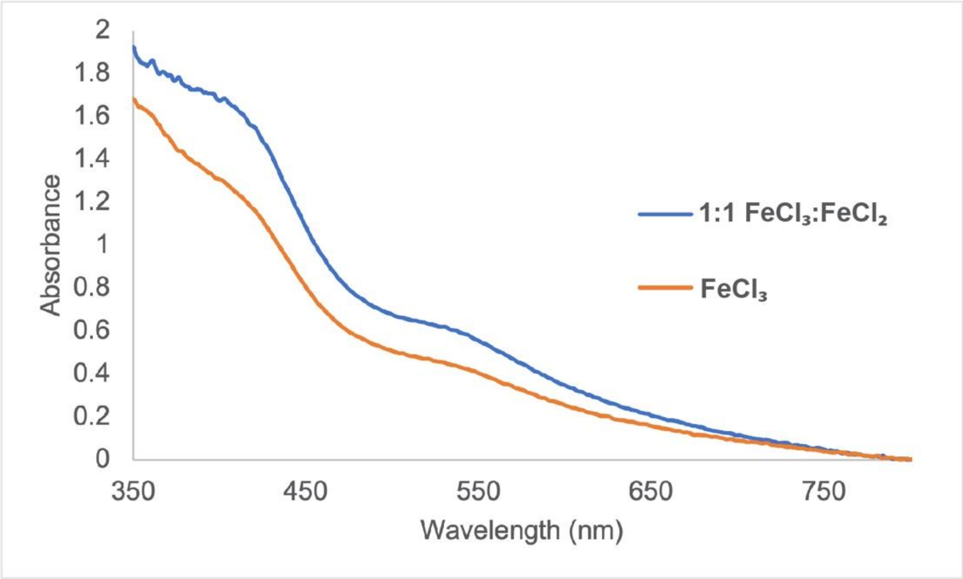

Supplementary Fig. 9. Representative UV-Vis spectrum for solution containing 5 mM Cysteine, 0.5 mM $FeCl_3$, 0.5 mM $FeCl_2$ and 1 mM Na_2S at pH ~9 (blue line). Spectrum of solution prepared with 5 mM L-cysteine, 1 mM $FeCl_3$ and 1 mM Na_2S at pH 9 also included for reference (orange line). Higher absorbance of mixed Fe solution is likely due to increased scattering from precipitate formation. Source data are provided as a Source Data file.

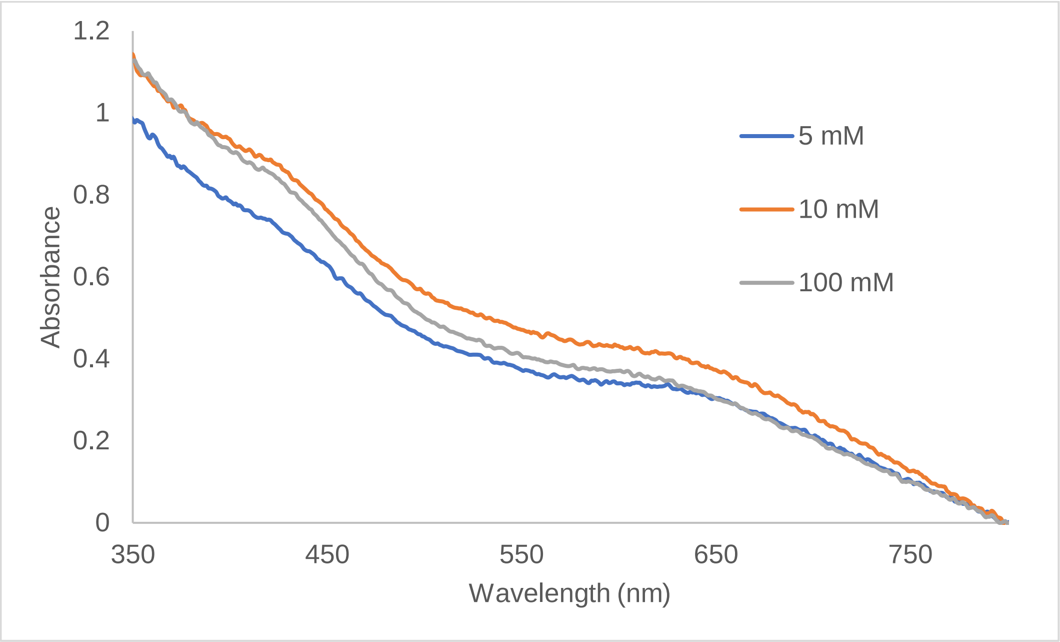

Supplementary Fig. 10. UV-Vis spectra for solutions of increasing $NaHCO_3$ concentration (5 to 100 mM) at pH ~9. $FeCl_3$ and Na_2S were both maintained at concentrations of 0.5 mM. Source data are provided as a Source Data file.

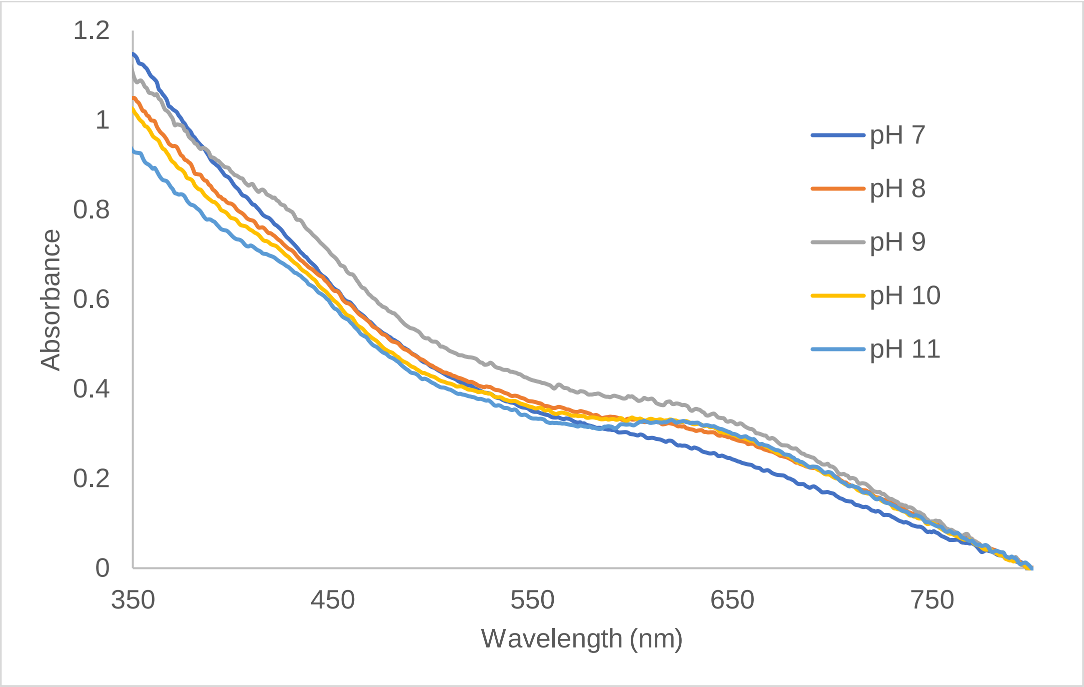

Supplementary Fig. 11. UV-Vis spectra for solutions containing 10 mM $NaHCO_3$, 0.5 mM $FeCl_3$, and 0.5 mM Na_2S across a pH range of ~7 to 11. Source data are provided as a Source Data file.

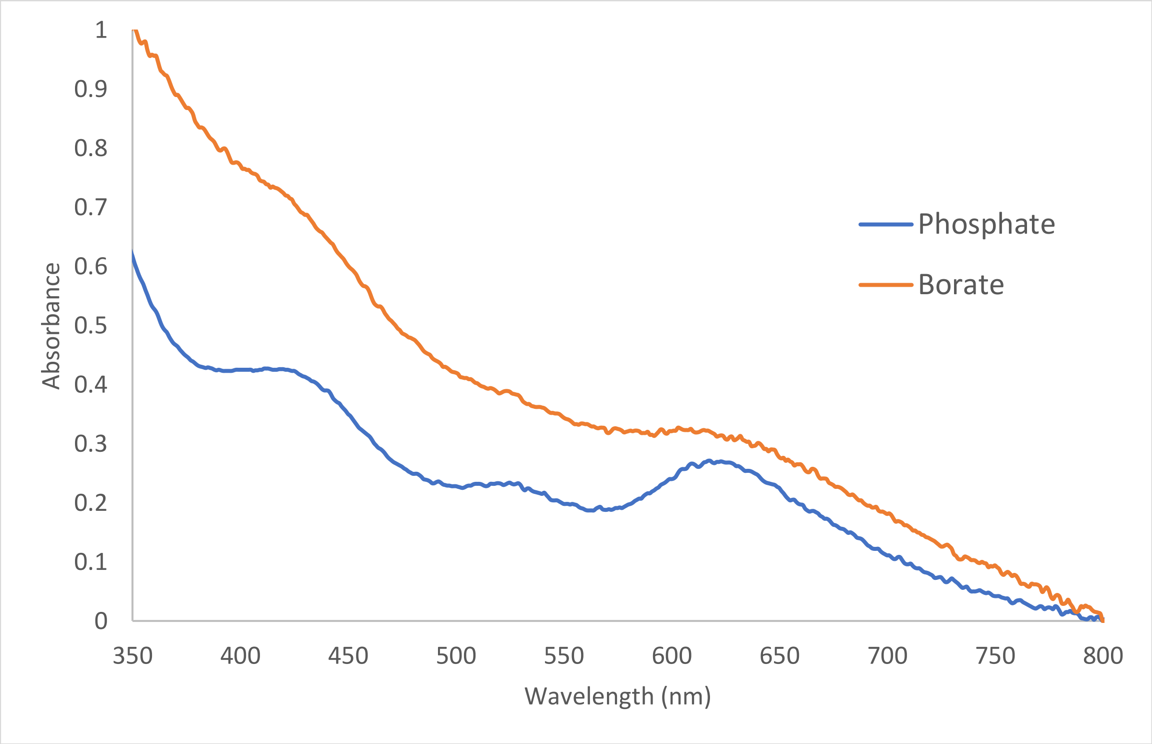

Supplementary Fig. 12. UV-Vis spectra for solutions of 0.5 mM $FeCl_3$ and 0.5 mM Na_2S prepared with phosphate (blue line - 80 mM Na_2PO_4) and borate (orange line - 40 mM $Na_2B_4O_7$) buffers. Source data are provided as a Source Data file.

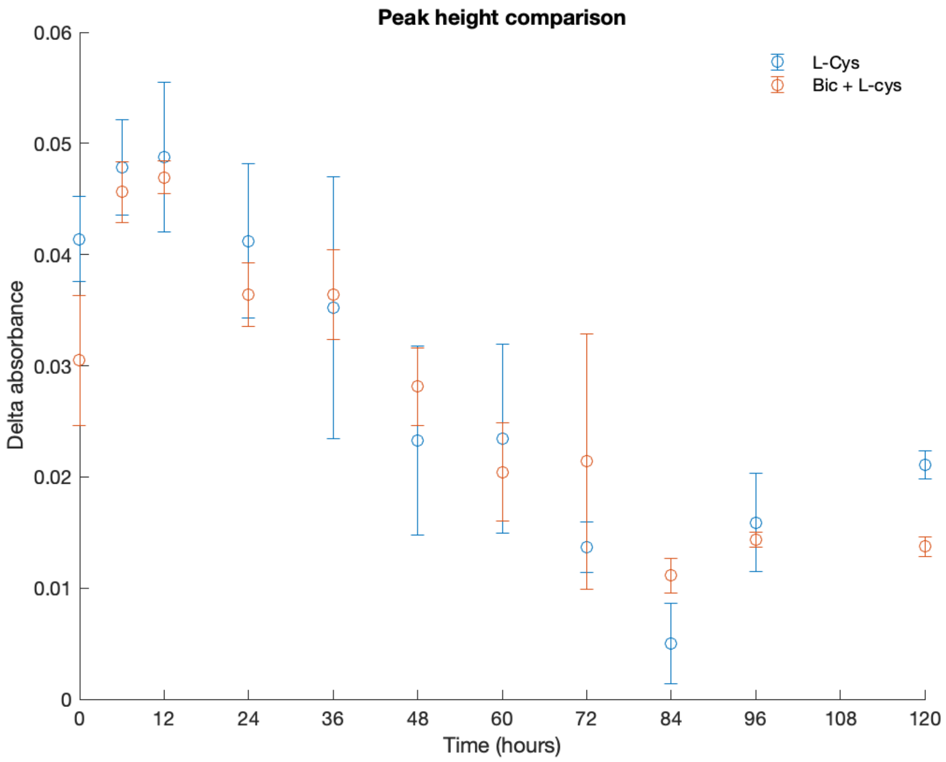

Supplementary Fig. 13. Evolution of the extracted 420 nm peak amplitude representative of clusters formed in the absence (blue) or presence (orange) of 10 mM $NaHCO_3$ over a 5-day period. Cys-FeS were prepared with 5 mM L-cysteine, 1 mM $FeCl_3$ and 1 mM Na_2S at pH 9. N = 3; the error bars show standard deviation around the mean (circles). Source data are provided as a Source Data file.

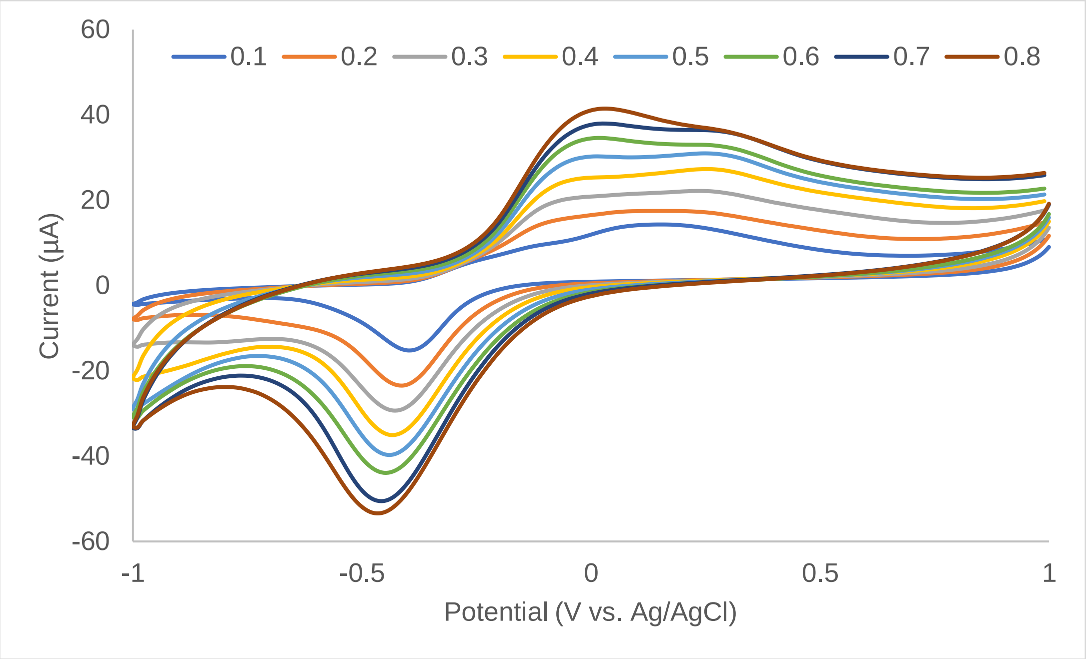

Supplementary Fig. 14. Cyclic voltammograms with increasing scan rate (0.1 to 0.8 V/s) for clusters formed with 3.5 mM L-cysteine, 1 mM $FeCl_3$ and 1 mM Na_2S at pH 9. Source data are provided as a Source Data file.

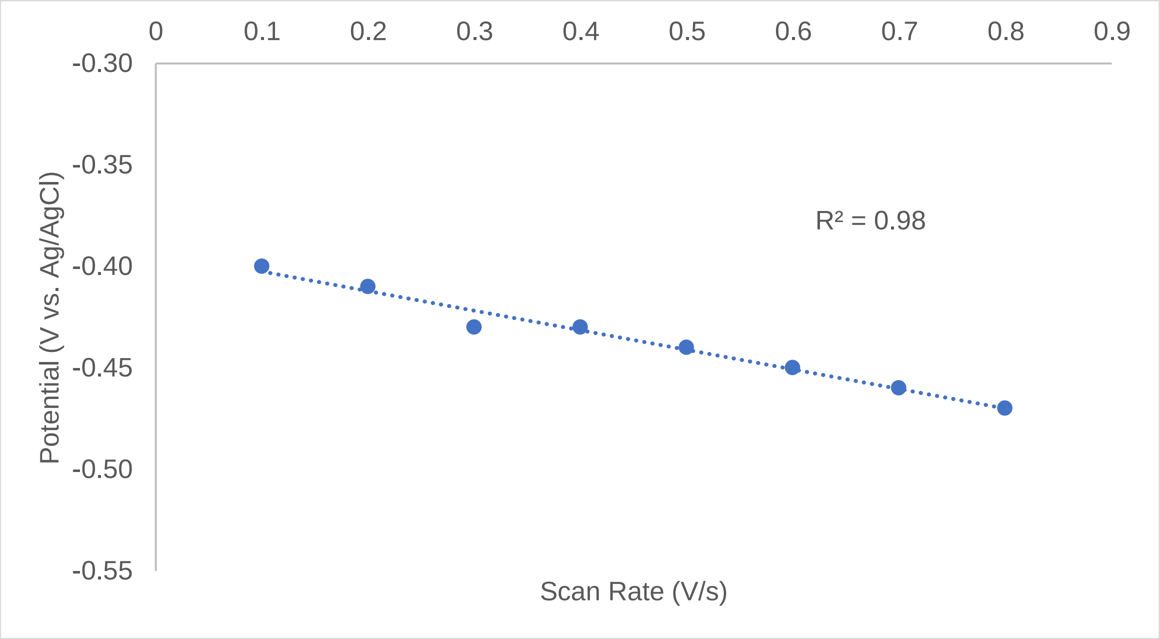

Supplementary Fig. 15. Potential vs. scan rate for reduction peak of clusters formed with 3.5 mM L-cysteine, 1 mM $FeCl_3$ and 1 mM Na_2S at pH 9 displaying a linear shift. Source data are provided as a Source Data file.

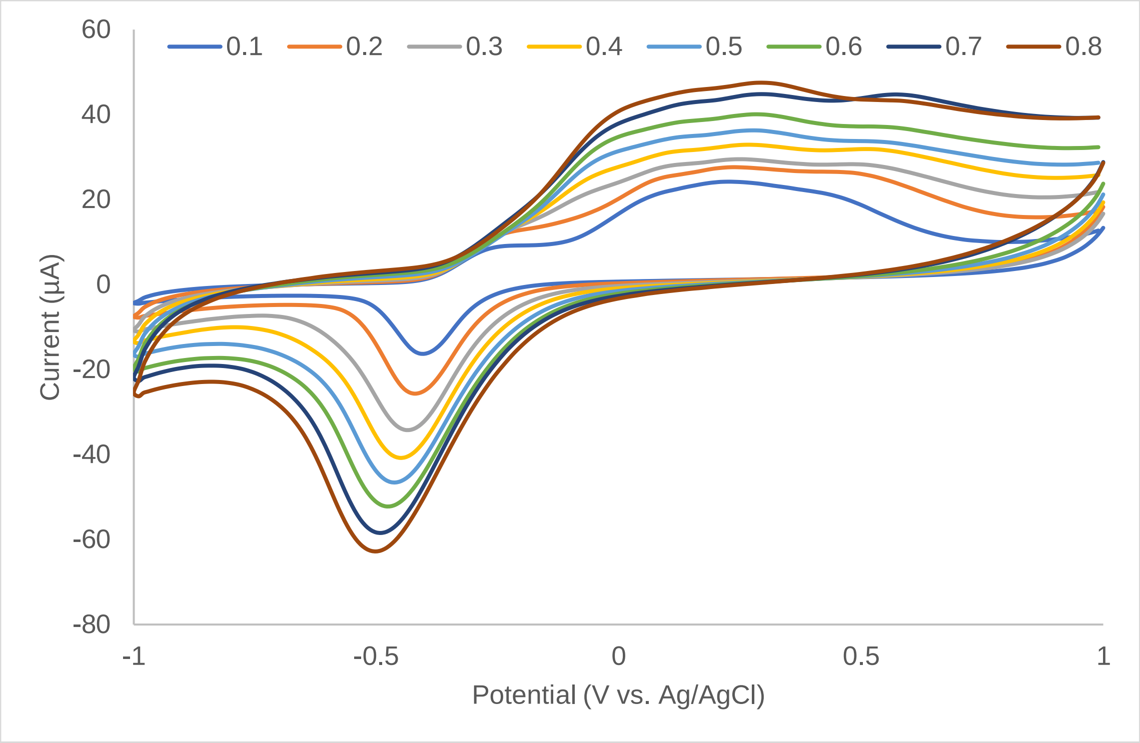

Supplementary Fig. 16. Cyclic voltammograms with increasing scan rate (0.1 to 0.8 V/s) for FeS clusters formed with 5 mM L-cysteine, 1 mM $FeCl_3$ and 1 mM Na_2S at pH 9. Source data are provided as a Source Data file.

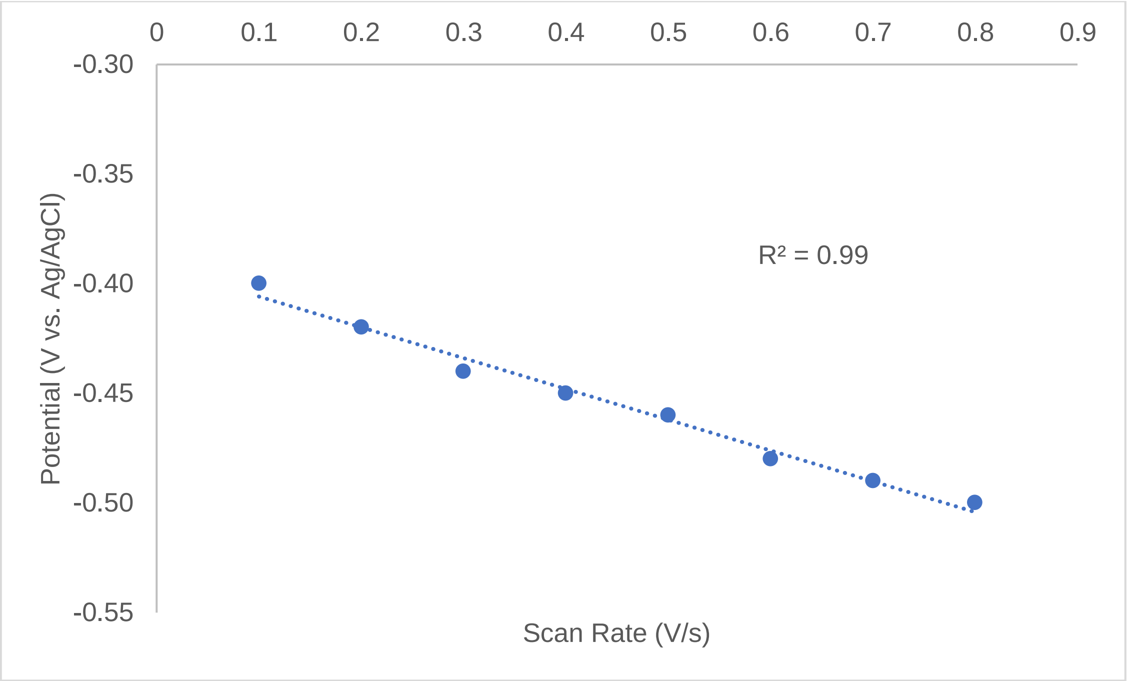

Supplementary Fig. 17. Potential vs. scan rate for reduction peak of FeS clusters formed with 5 mM L-cysteine, 1 mM $FeCl_3$ and 1 mM Na_2S at pH 9 displaying a linear shift. Source data are provided as a Source Data file.

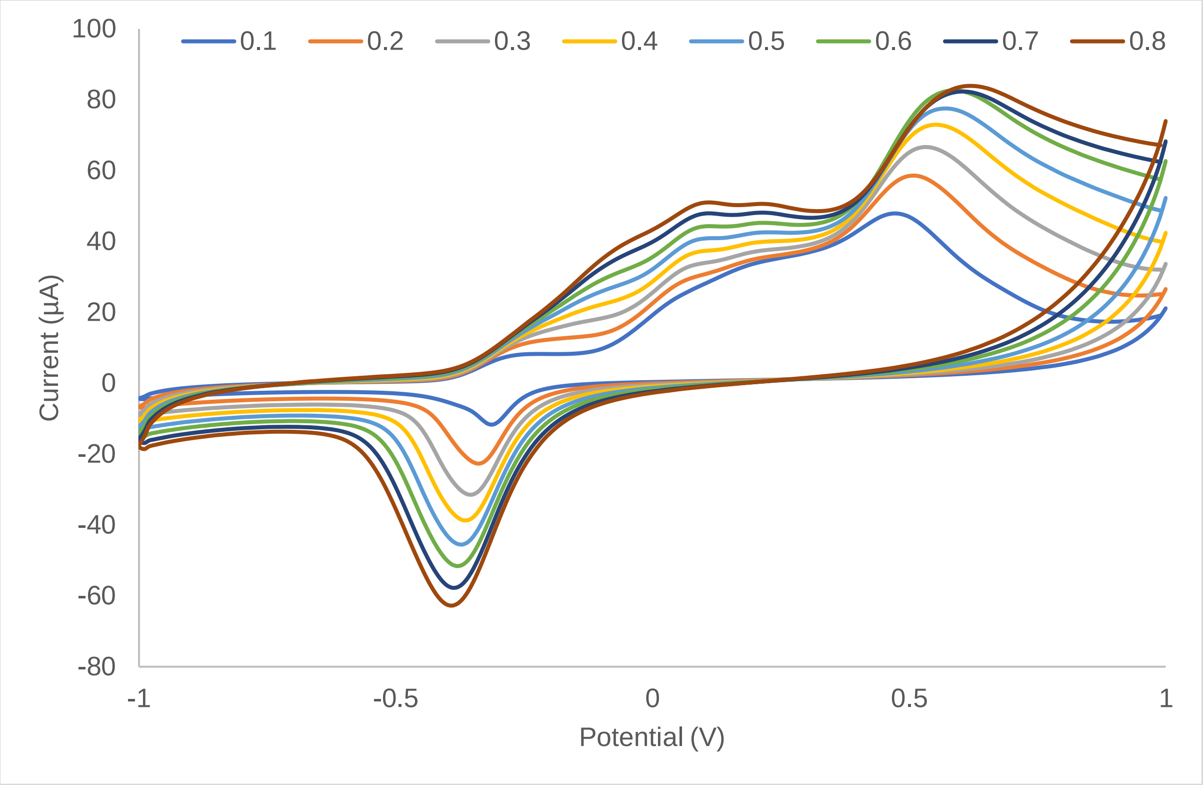

Supplementary Fig. 18. Cyclic voltammograms with increasing scan rate (0.1 to 0.8 V/s) for FeS clusters formed with 10 mM L-cysteine, 1 mM $FeCl_3$ and 1 mM Na_2S at pH 9. Source data are provided as a Source Data file.

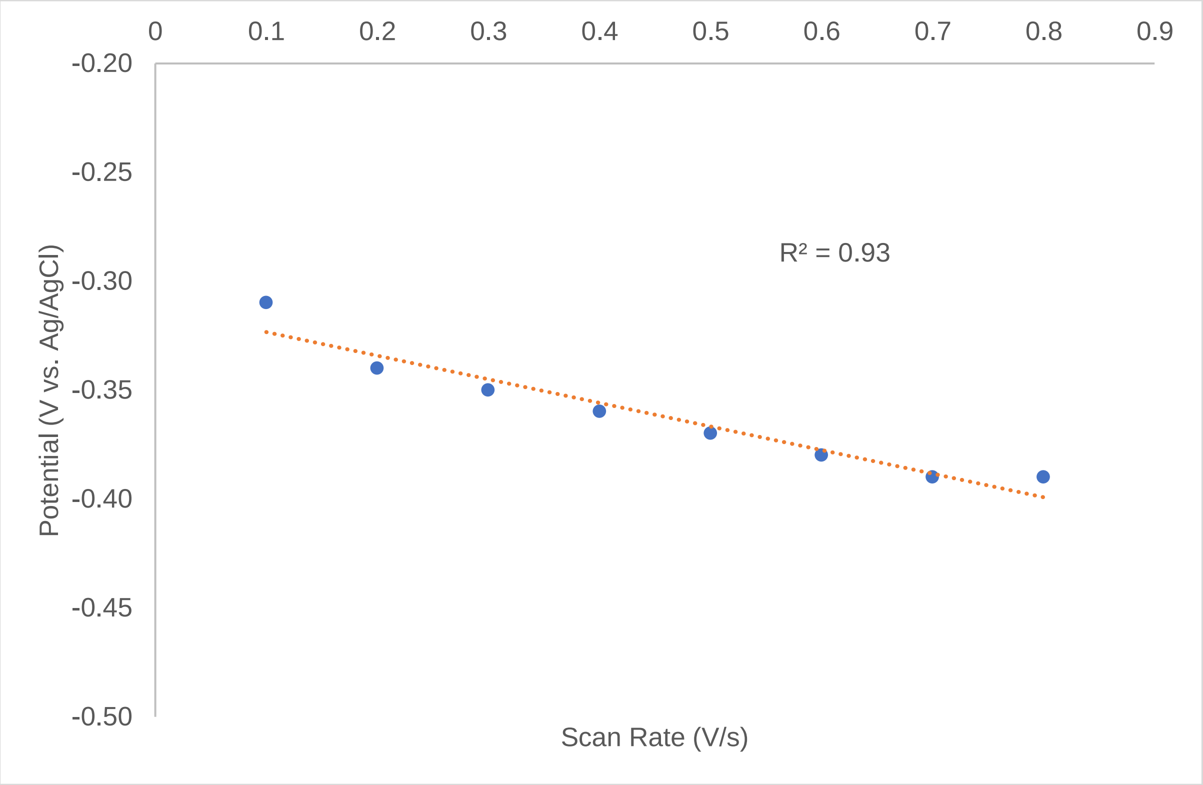

Supplementary Fig. 19. Potential vs. scan rate for reduction peak of FeS clusters formed with 10 mM L-cysteine, 1 mM $FeCl_3$ and 1 mM Na_2S at pH 9 displaying a non-linear shift. Source data are provided as a Source Data file.
